# How to improve the equity of health financial sources? - Simulation and analysis of total health expenditure of one Chinese province on system dynamics

**DOI:** 10.1186/s12939-015-0203-x

**Published:** 2015-08-27

**Authors:** Xin Wang, Yuanling Sun, Xin Mu, Li Guan, Jingjie Li

**Affiliations:** Department of the Health Service Management, China Medical University, Executive director of China Health Economic Association, Director of China Soft Science Association. Major Research on: health economic and policy, No.77 Puhe Road, Shenyang North New Area, Shenyang, Liaoning Province 110122 China (PRC); China Medical University, No.77 Puhe Road, Shenyang North New Area, Shenyang, Liaoning Province 110122 China (PRC); Department of Humanities and Social Sciences, China Medical University, No.77 Puhe Road, Shenyang North New Area, Shenyang, Liaoning Province 110122 China (PRC); Shenyang Medical College, No.146 Huanghe bei Road, Huanggu District, Shenyang city, Liaoning Province 110032 China (PRC); Department of the Health Service Management, China Medical University, No.77 Puhe Road, Shenyang North New Area, Shenyang, Liaoning Province 110122 China (PRC)

## Abstract

**Introduction:**

We simulate and analyze Total Health Expenditure (THE) in financial sources and other economic indicators (such as THE per capita, GDP, etc.) in a province of China from 2002 to 2012 on System Dynamics.

**Methods:**

Based on actual data and certain mathematical methods, we use system dynamic software to construct a logic model for THE and changing proportions, and thus simulate the actual conditions of development and changes in THE.

**Results:**

According to the simulation results, the government possess the largest investment in the average annual growth rate of THE, which was 25.16 % in 2012. Social investment comprises the majority of the possession ratio, which was up to 41.20 %.

**Conclusions:**

The personal investment growth rate decreased by almost 21 %, but the total amount of personal investment increased by 28075 million yuan, which is far higher than the increase in government investment. Individuals are still the main carriers of health care expenses. The equity of health financial sources is still poor. The System Dynamics method used in this paper identifies a dynamic measurement process, provides a scientific basis for simulation and analysis of the changes in THE and its key constraining factors, as well as put forward suggestions for the improvement of equity of health financial sources.

## Introduction

In recent years, health care has become a hot issue of China's social and economic development, especially the severe health expenditure growth situation, which not only makes the government face enormous financial pressure but also throw more pressure on individual residents’ expenses. Recently, international organizations and many countries have paid great attention on researches of total health expenditure. Rising cost of health care has been identified as an enormous obstacle and challenge all over the world [1]. The challenge that less developed countries are facing is to maintain healthy services under the situation of global economic recession and the changeable economic structures; What’s more, the rich countries try to control the growth rate of medical expenses not to exceed that of Gross Domestic Product (GDP) [2, 3]. However according to national health account studies, in the meantime China’s health care costs has grown much faster than the economic growth by far [4].

Not only the total amount of the total health expenditure is increasing, the total expenditure on health financing and distribution structure is unfair. World Health Organization announced that all members of the society should have equal access to basic health services, which means that all residents should enjoy basic health services at the same level [5]. Since 2003, China has gradually introduced health reform, including innovative financing of health care, such as: increased government spending on health, the introduction of new medical insurance (such as basic medical insurance system for urban residents and the new rural cooperative medical system), and the expansion of health insurance coverage [6].

However, there is still a big difference between the levels of financing and security of different medical insurance systems. Such as the big gap between urban and rural medical security system. The security levels of the same system in different areas are also different; In 2011, the per capita medical expenses of urban households in China is 969 yuan, which is more than 2 times per capita medical expenses in rural families (436.8 yuan). The regional distribution of the total health expenditure is also seriously unbalanced. Such as medical expenses of urban residents, the highest in Beijing (1327.2 yuan) is 2.43 times to the lowest in Guizhou (546.8 yuan); Medical expenses of rural residents in Beijing (840.6 yuan) is 4.72 times to Guizhou (178.1 yuan). These have led to the internal unfair of health financing structure,which makes the residents’ personal cash health spending too high.

To realize the structural adjustment to THE, the common characteristics of health services need to be highlighted and the level of health protection of society as a whole has been raised [7]. Many countries’ reform goal is to improve the health financing equity. However, how to improve the equity? Financing equity can explore the problems of health financing structure in China, and to find effective suggestions to solve these problems.

Total health expenditure is an important part of GDP, refers to total capital expenditure of a country or region's health services in a given period, which reflects the financial level and degree of utilization of health insurance financing [8]. Total health resources are composed of the total amount for raising or spending of health resources. According to this source, THE includes Government Health Expenditure (GHE), Social Health Expenditure (SHE) and Out-of-Pocket health expenditure (OOP).

System Dynamics is a commonly used dynamic computer method for simulating system dynamic process, which builds a particular relationship of logical constraints. It mainly studies the dynamic characteristics of how the system's behavior changes over time, and it is a branch of systematic science and scientific management system [9]. System dynamics started late in China, but developed rapidly. Many scholars use this method to analyze a number of long-term and cyclical issues as well as social and economic problems which are more complex. Recently, the system dynamics has been widely used in a number of complex system fields of both natural and social sciences, but its usage in health sector is still in an early stage.

Based on System Dynamics, this paper compares and analyzes the relationship between the THE financial resource, changing characteristics, fiscal expenditure and GDP in a province from 2002 to 2012.

## Materials and methods

### Data

The research data comes primarily from the annual results of the THE for Liaoning province (by Liaoning Health Bureau and Liaoning Health Economy Acad.), the Liaoning Statistical Yearbook, the statistical bulletin of Liaoning economic and social development and etc. The research data was obtained following the protocol approved by Ethics Committee of China Medical University.

### Tool

We use the software of STELLA 9.1 on System Dynamics, which is a modeling tool produced by ISEE in the United States. With the reference to original data, we designed a simulation process that uses mathematical formula and data transformation.

### Method and step

#### Structure and function of the model

**Fig. 1:**
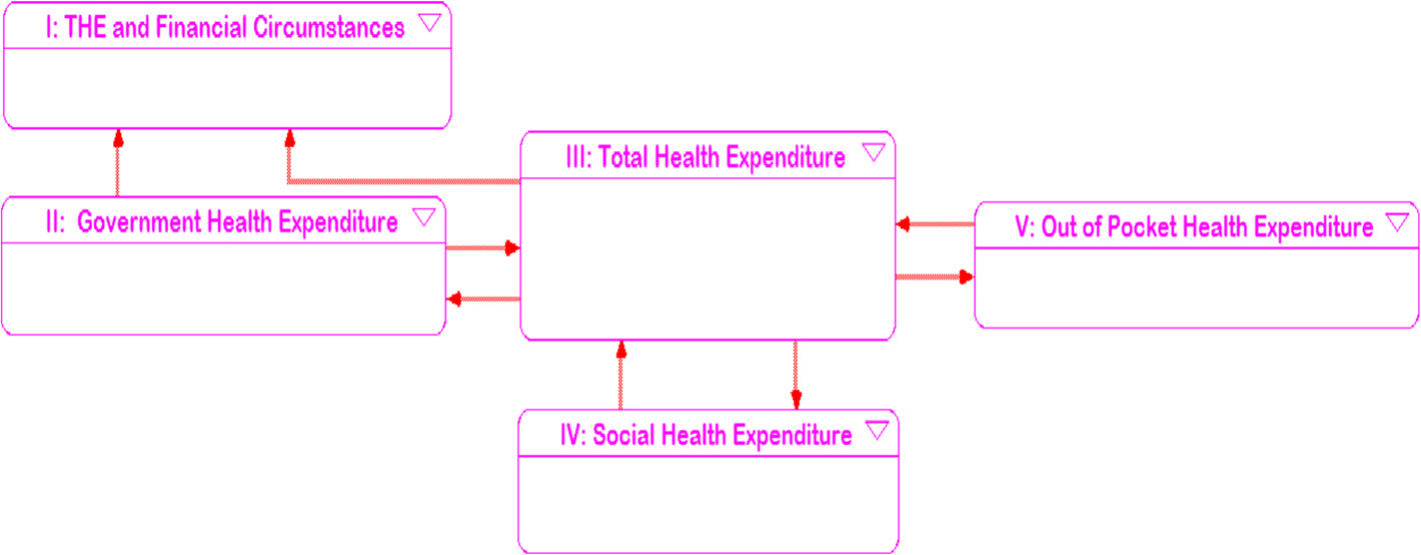
THE financial resource and related factors

We construct the structure model of composition of THE in Fig. 1 based on the THE financial resource and comparison with other economic indicators [10].

**Fig. 2:**
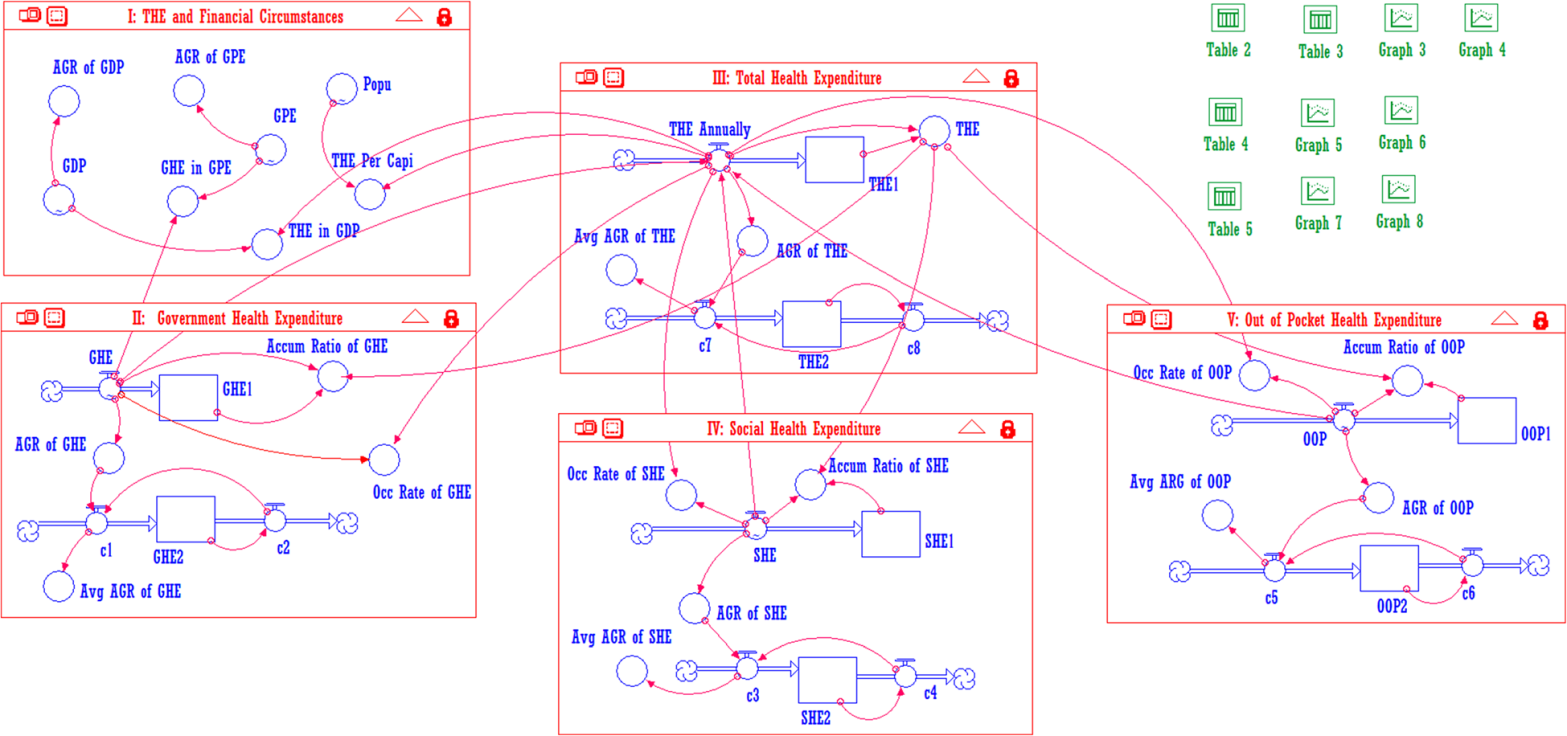
The THE financial resources portfolio process

The dismantling structure and the relationships between each module are shown in Fig. 2 (model IV and model V are the same as model II in structure). Model I (THE in GDP and Population) simulates indicators such as provincial government investment in THE per annum, the proportion of THE in GDP and THE per capita; model II (Expenditure by Government), model IV (Expenditure by Organizations) and model V(Expenditure by Individuals) simulates the proportion of GHE, SHE, OOP in THE, the growth rate, and the average growth rate; model III (Total Health Expenditure) demonstrates changes in the THE per annum, including the annual growth rate, the average annual growth rate and the cumulative amount year by year.

The main data elements, abbreviations and interpretations used in the models are as follows (Table 1):

**Table 1:** Abbreviations for major data names and interpretations

THE	Total health expenditure	AGR	Annual growth rate
GDP	Gross Domestic Product	GPE	Government Public Expenditure
GHE	Government Health Expenditure	Popu	Population of Province
SHE	Social Health Expenditure	Accum Ratio	Accumulative Ratio
OOP	Out-of-Pocket Health Expenditure	Occ Rate	Occupancy Rate

#### The original data table

After building the model, we input the data of THE financial resource and other related economic data into the table. The simulation of original data table is as follows (Table 2):

**Table 2:** The original data related to THE (Unit: million CNY or million capita)

Years	GHE	SHE	OOP	THE annually	GDP	GPE	Popu
2002	2, 470.29	5,906.50	11,420.00	19,796.79	545,800.00	69,210.00	41.55
2003	3, 054.91	8,543.80	12,970.00	24,568.71	600,300.00	78,460.00	41.62
2004	3,531.57	9,091.49	14,667.52	27,290.58	687,290.00	92,060.00	41.73
2005	4, 866.70	11,402.90	21,440.80	37,710.40	800,900.00	118,227.00	41.89
2006	5,834.83	14,621.35	21,547.31	42,003.49	925,120.00	141,857.00	42.10
2007	6,601.19	18,152.12	23,960.03	48,713.34	1,102,350.00	176,428.00	42.32
2008	8,490.12	22,660.42	25,371.20	56,521.74	1,346,160.00	215,343.00	42.46
2009	14,600.09	26,267.51	30,612.43	71,480.03	1,521,000.00	268,239.00	42.56
2010	17,632.00	30,162.00	32,704.00	80,498.00	1,845,730.00	319,582.00	43.75
2011	21,016.45	32,260.38	35,285.56	88,562.39	2,222,670.00	390,585.00	43.83
2012	23,302.44	44,005.87	39,495.36	106,803.67	2,484,640.00	455,859.00	43.89
Final					2,484,640.00	455,859.00	43.89

#### Simulating process

The original table in model II transfers the GHE data to the accumulator GHE1 and converts AGR of GHE respectively, as well as calculates the cumulative results of GHE per annum and the annual growth rate. Then it imports the annual growth rate into the loop structures to determine the average growth rate per year. In model III, we use the data GHE, SHE and OOP from model II, IV, V to calculate the THE per year, then use the previously mentioned processes to calculate the cumulative THE, growth rate, and average annual growth rate. At the same time, the data converter outputs the cumulative annual THE to module I, II, IV and V. Using the corresponding data elements like Popu, GHE, SHE and OOP, we calculate THE per capita each year, and the percentage of GHE, SHE and OOP. In summary, the entire model simulates 21 related indicators and provides a dynamic analytical method for describing and analyzing THE and the related factors [11].

## Results and analysis

### Changes in the THE financial resource and cumulative share of various sources per annum

According to the procedure above, the simulation results are as follows (Table 3):

**Table 3:** The occupancy rate of GHE, SHE and OOP (Unit: percent or million CNY)

Years	Occ rate of GHE	Occ rate of SHE	Occ rate of OOP	THE annually	Accum ratio of GHE	Accum ratio of SHE	Accum ratio of OOP	THE
2002	12.48	29.84	57.69	19796.79	12.48	29.84	57.69	19796.79
2003	12.43	34.78	52.79	24568.71	12.45	32.57	54.98	44365.50
2004	12.94	33.31	53.75	27290.58	12.64	32.85	54.51	71656.08
2005	12.91	30.24	56.86	37710.40	12.73	31.95	55.32	109366.48
2006	13.89	34.81	51.30	42003.49	13.05	32.74	54.20	151369.97
2007	13.55	37.26	49.19	48713.34	13.17	33.84	52.98	200083.31
2008	15.02	40.09	44.89	56521.74	13.58	35.22	51.20	256605.05
2009	20.43	36.75	42.83	71480.03	15.07	35.55	49.37	328085.08
2010	21.90	37.47	40.63	80498.00	16.42	35.93	47.65	408583.08
2011	23.73	36.43	39.84	88562.39	17.72	36.02	46.26	497145.47
2012	21.82	41.20	36.98	106803.67	18.45	36.94	44.62	603949.14
Final						37.58	43.47	

**Fig. 3:**
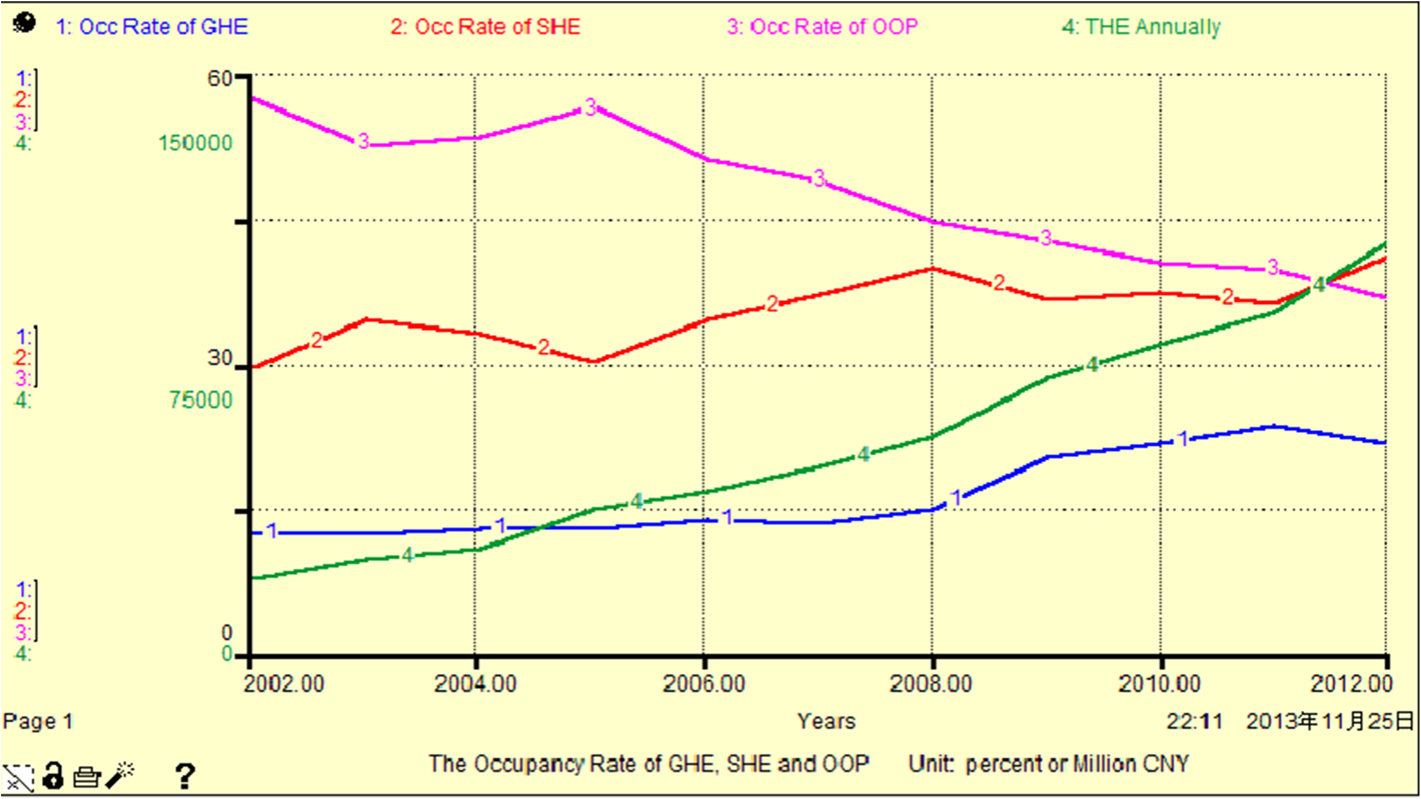
The occupancy rate of GHE, SHE and OOP (Unit: percent or million CNY)

**Fig. 4:**
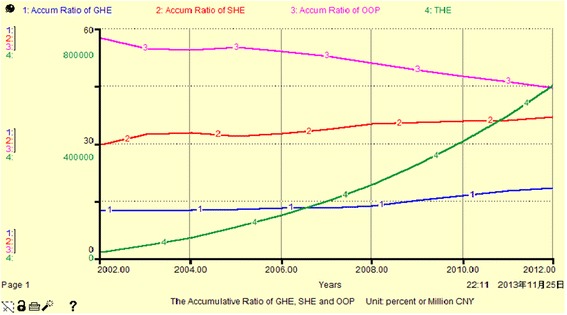
The accumulative ratio of GHE, SHE and OOP (Unit: percent or million CNY)

Figure 3, Fig. 4 and Table 3 show that the share of THE financing sources and the share of cumulative THE financing sources are between 12 % and 58 %. Among them, the GHE share is the smallest, having increased to 21.82 % in 2012, OOP is the largest with a peak of 57.69 % in 2002. The changes in GHE and SHE show a slow increasing trend over the years, with a total increase of approximately 8–10 %. However, OOP is gradually decreasing by approximately 21 % totally. This indicates that the government and community institutions have gradually reduced the proportion of personal investment in THE since 2002. From another aspect, the cumulative THE is 19796.79–603949.14 million yuan from 2002 to 2012, but the cumulative GHE accounts for 18.45 %, and this indicates that because of the low original investment and low annual increment, the main contributors to THE are still individuals and social institutions.

### The THE growth rate and average growth rate of all sources per annum

The following table and figures show the simulation results (Table 4):

**Table 4:** The Ave and AGR of parts and whole of Health Expenditure (Unit: percent)

Years	AGR of GHE	AGR of SHE	AGR of OOP	AGR of THE	Avg AGR of GHE	Avg AGR of SHE	Avg AGR of OOP	Avg AGR of THE
2002	0.00	0.00	0.00	0.00	0.00	0.00	0.00	0.00
2003	23.67	44.65	13.57	24.10	23.67	44.65	13.57	24.10
2004	15.60	6.41	13.09	11.08	19.57	24.07	13.33	17.41
2005	37.81	25.42	46.18	38.18	25.36	24.52	23.36	23.96
2006	19.89	28.22	0.50	11.38	23.97	25.43	17.20	20.69
2007	13.13	24.15	11.20	15.97	21.72	25.18	15.97	19.73
2008	28.61	24.84	5.89	16.03	22.85	25.12	14.23	19.11
2009	71.97	15.92	20.66	26.46	28.89	23.76	15.13	20.13
2010	20.77	14.83	6.83	12.62	27.85	22.61	14.06	19.17
2011	19.19	6.96	7.89	10.02	26.86	20.76	13.35	18.11
2012	10.88	36.41	11.93	20.60	25.16	22.24	13.21	18.36
Final								

**Fig. 5:**
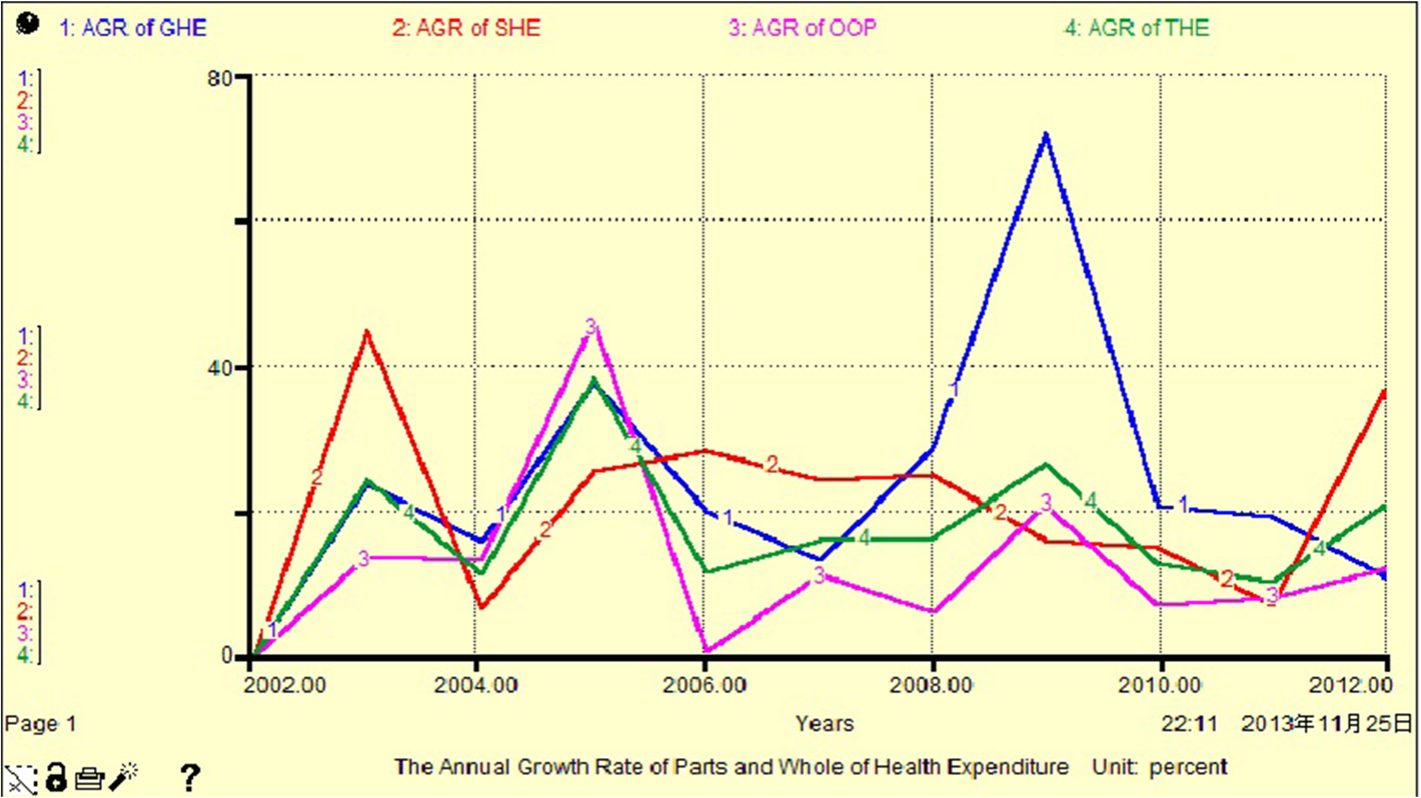
the AGR of GHE, SHE, OOP and THE (Unit: percent)

**Fig. 6:**
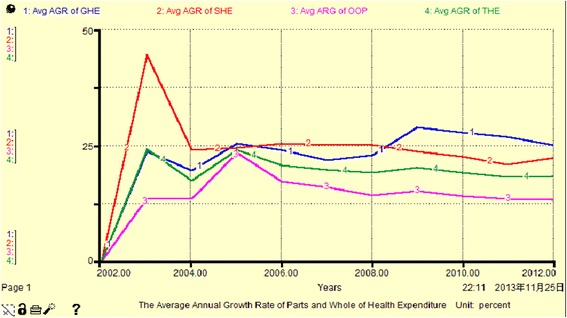
The Avg AGR of GHE, SHE, OOP and THE (Unit: percent)

From Fig. 5, Fig. 6 and Table 4, we see that the THE growth rate per annum for all sources has high volatility, between 0.5 % and 72 %. Among these, the annual growth rate of GHE exhibits positive fluctuation close to or higher than that for THE (other than in 2007), and has reached a peak value of 71.97 % in 2009. We can divide the period 2002–2012 into three sub periods with respect to the average annual growth rate of THE. The GHE was slightly higher than the SHE (less than 1 %) in 2005, and OOP had the biggest increase (more than 10 %). SHE ranked first from 2006 to 2008, and reached 25.12 % in 2008, while GHE comprised 25.16 % in 2012. Overall, SHE maintained a stable rate of increase, essentially replacing GHE and playing a dominant role in THE. The percentage of OOP is not reduced, due to fluctuations in GHE and SHE, it changes equidirectionally. This indicates that GHE, SHE and OOP do not have complementary mechanisms.

### Integrated features of THE and related financial indicators

Integrating the previously mentioned THE data and using the related economic indicators, the following results are obtained (Table 5):

**Table 5:** The indexes about health expenditure and financial circumstances (Unit: percent/CNY)

Years	AGR of THE	AGR of GHE	Occ rate of OOP	AGR of GDP	AGR of GPE	GHE in GPE	THE in GDP	THE Per Capi
2002	0.00	0.00	57.69	0.00	0.00	3.57	3.63	476.46
2003	24.10	23.67	52.79	9.99	13.37	3.89	4.09	590.31
2004	11.08	15.60	53.75	14.49	17.33	3.84	3.97	654.01
2005	38.18	37.81	56.86	16.53	28.42	4.12	4.71	900.18
2006	11.38	19.89	51.30	15.51	19.99	4.11	4.54	997.61
2007	15.97	13.13	49.19	19.16	24.37	3.74	4.42	1,151.15
2008	16.03	28.61	44.89	22.12	22.06	3.94	4.20	1,331.14
2009	26.46	71.97	42.83	12.99	24.56	5.44	4.70	1,679.51
2010	12.62	20.77	40.63	21.35	19.14	5.52	4.36	1,839.95
2011	10.02	19.19	39.84	20.42	22.22	5.38	3.98	2,020.59
2012	20.60	10.88	36.98	11.79	16.71	5.11	4.30	2,433.44
Final				0.00	0.00			

**Fig. 7:**
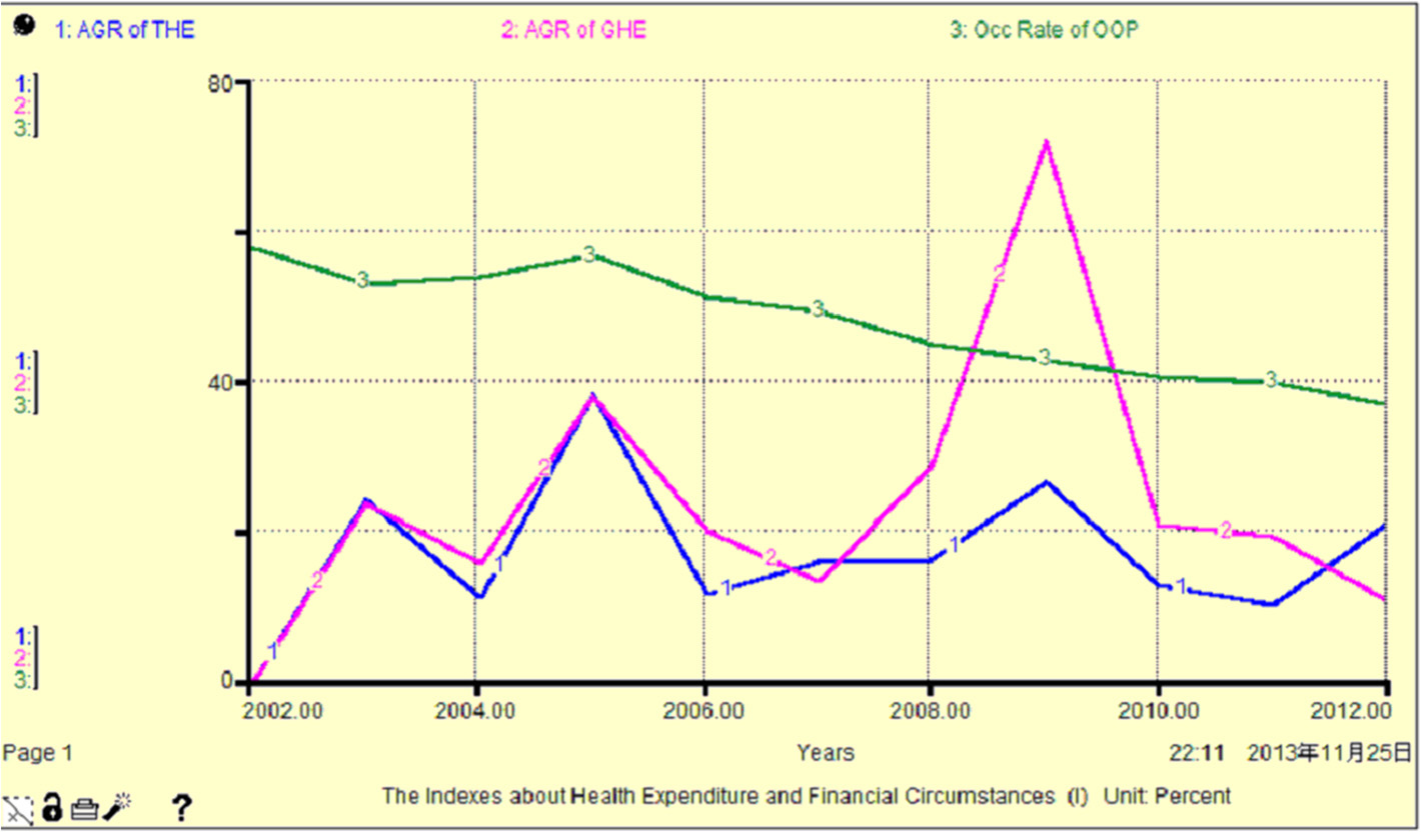
Simulation map I of THE and financial indicators (Unit: percent)

**Fig. 8:**
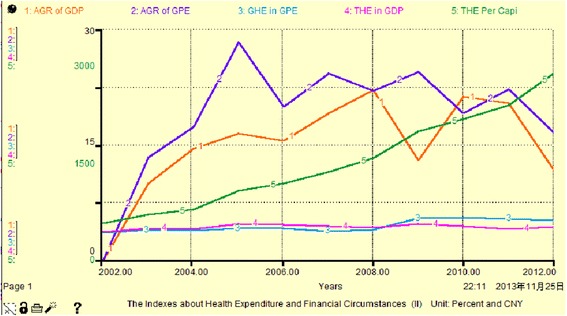
Simulation map II of THE and financial indicators (Unit: percent and CNY)

From Fig. 7, Fig. 8 and Table 5, we observe that financial expenditure fluctuation over these years is generally increasing. Thus GHE and THE also change with the same characteristics. Government improved their investment in health in 2008, which caused an obvious increase in THE. Only in 2006 and 2007,China's central government has increased the health budget by 87 % [12]. However, because the proportion of GHE in general financial expenditure is too low (less than 6 %) and is much less than half of the THE, even less than SHE and OOP (see Table 5), we find that the investors in THE are mostly social institutions and individuals.

From Table 5, the expansion of GHE reduced the proportion of OOP in the THE from 58 % to approximately 37 %. THE per capita also increased to 2433 Yuan (five times the original value) during the same period. However, this is still far from the ideal proportions (15–20 %) proposed by the World Health Organization for non-family financial burden. In addition, the large increase in THE has not produced any obvious changes in the proportion of GDP because of the low proportion of GDP (<5 %). Increasing GDP without THE would be unilateral and not lead to good development or patterns for the overall social health of the area. China's experience shows that the development of health and economy are not fully synchronized [13].

## Discussion

### Comprehensive analysis and comparison

From the above data on the growth of THE, GHE contributed the most in 2012 at 25.16 %, SHE come in the second with 22.24 %, and OOP contributed the least at 13.21 % (see Table 4). As many other developing countries, the main sources of financing health care in China is central and local government funds, employer-paid medical insurance for urban workers as well as individual cash payments [4]. However, from the perspective of capital scale we see the opposite pattern. In 2012, for example, GHE had the smallest share at only 21.82 %, OOP was in the middle with 36.98 %, and SHE was the largest with, 41.20 %. Their cumulative share spreads were between 18.45 % and 44.62 % (see Table 3). The difference in total investment also confirmed the burden on personal investment (see Table 2). The growth in OOP is nearly 28075 million yuan (11420.00–39495.36), and social investment has increased nearly 38099 million yuan (5906.50–44005.87), while government investment has increased by only 20832 million yuan (2470.29–23302.44) (see Table 2). Therefore, while the decline in the relative growth rate of OOP obscures the absolute growth in the total amount, individuals are still the main contributors to THE. It suggests that the equity of health financial sources in China is still poor.

In Asia, cash payment is the primary means of health care financing ; due to the uncertainty of medical expenses reduces the welfare of residents, families can borrow money to pay for unexpected medical expenses, but the uncertainty of medical expenses may result in long-term debt [14]. OOP spending is an inefficient method in financing health [15]. It could have a negative impact, including in fairness and making people poor. Several studies show that, for developing countries, a high share of OOP has serious consequences in health financing. Financial barriers have become the largest obstacle for the poor accessing to health care [16]. OPP accounted for a higher proportion of Total Health Expenditure is an important factor to cause unfairness. The amount of GHE investment has been increasing each year, but due to the low original level, the overall increase is limited.

From the indicators related to THE, the share of THE in GDP and GHE in GPE in 2012 were 4.30 % and 5.11 % respectively (see Table 5). The former is lower than the national level (5.36 %), while the latter is also lower than the national level (5.36 %). The ratio of total health expenditure to GDP is an important indicator of the international and domestic evaluation of the total health expenditure, reflecting whether the total health expenditure and GDP can achieve coordinated development.

### Research and application of System Dynamics of THE

The health system is a combination of function and structure. It is operating in the social economy, population and environment factors. The factors are related to each other. Therefore, the use of conventional methods in the trend of total health expenditure projections is a big difficulty [17]. System Dynamics is a commonly used method for simulating dynamically changing processes that involve computer systems. It builds up logical restriction relations between the principal factors within the system, showing and evaluating the running features and validity of elements in the system. By using System Dynamics in THE to compare various sources analyze the linkages with other economic indicators, and by analyzing the interaction of systems, our results emphasized the “spot to area” description of the internal process. Thus it can be an effective method for studying the economic structure of health. Therefore, using system dynamics theory and model research can provide new methods and tools to calculate the total health expenditure.

System dynamics model projections of total health expenditure have advantages: first, more scientific. System Dynamics and traditional econometric methods, which are based on information feedback principle, more attention on causal relationship between variables, structural characteristics of the model to solve the problem directional accuracy and system operation. Therefore, it is more suitable for this complex health system. Second, it has no strict requirement for the distribution of the data. System Dynamics is not strictly dependent on accurate data and time series, emphasizing the relationship of internal and external factors during the dynamic changes in the system embodied in the development of process, which is more conducive to multi-factor analysis of total health expenditure. The third result is easy to implement. System dynamics theory after 50 years of development and exploration has developed a variety of process intuitive and easy adjustment of computer-aided inspection software. In short, the system dynamics not only improves computational efficiency, but also makes the results more accurate and reliable.

## Conclusions

We think the increase of Out-of-Pocket health expenditure has always had a close relationship with the transformations of the urban and rural income levels increase and consumer attitudes. However, if it is caused by excessive medical and drug costs, we should pay more attention to that nonprofit health care institutions should be maintained, to avoid false drug expenses which increases unreasonably. So to solve to problem of high pocket health expenditures , we can not just start from the control of the total amount of the cost of health, but also from health financing structures that were analyzed. On the adjustment of financing structure, ideal goal of personal health spending share should be below 30 %.

We put forward some policy suggestions to reduce the proportion of Out-of-Pocket health expenditure and to set up a multi-level health care financing system in China, in order to enhance the equity of health financing. We suggest that public financing (ie, government and social health insurance expenditures) should be the leading part of health financing. Financing of Health in our country is the mixed system of government's tax and social insurance. The relatively weak economy, large population, health reforms starting late and other objective conditions, make a single health financing not meet the demand of our health. So China takes a variety of funding models. According to the theory of welfare economics, public financing is conducive to social and economic risk sharing disease. Generally speaking, the larger proportion of public financing is, then the more equitable health financing will be.

World Health Organization reported that using of tax financing of health insurance or funds, through appropriate means to ensure co-ordination and implementation of services and sharing economic risks, reducing individual direct billing of medical services. With economic growth, China plans to invest an additional $25–38 billion government investment funds, equivalent to 1 %– 1.5 % of GDP [18]. We recommend that the government should increase investment in health, while emphasizing the need to run the regulatory capital and usage.

China government should implement the compensation policy of basic health finance,and change the past finance by local government investment to the central government as the main financial subsidies. In particular, less developed regions should be invested by the central government. Social aspects should encourage social capital to deal with medical institutions to increase the proportion of social spending on health.

Over time, the government and social institutions should cooperate with each other to coordinate and increase investment rates, significantly reduce the proportion of pocket health expenditure and achieve structural adjustment of health expenditure. The ultimate goal is to highlight the characteristics of public welfare and health services and to improve the level of health equity of the whole society.

## Abbreviations

THE: Total health expenditure
GDP: Gross domestic product
GHE: Government health expenditure
SHE: Social health expenditure
OOP: Out-of-pocket health expenditure
AGR: Annual growth rate
GPE: Government public expenditure
Popu: Population of Province
Accum Ratio: Accumulative Ratio (Definition:The cumulative ratio, refers to the proportion of GHE, SHE and OOP to THE, after the cumulative amount year by year of GHE, SHE and OOP)
Occ Rate: Occupancy Rate (Definition:to the ratio of GHE, SHE and OOP to total health Expenditure (THE))
